# Pediatric outpatient utilization by differing Medicaid payment models in the United States

**DOI:** 10.1186/s12913-020-05409-w

**Published:** 2020-06-12

**Authors:** Therese L. Canares, Ari Friedman, Jonathan Rodean, Rebecca R. Burns, Deena Berkowitz, Matt Hall, Elizabeth Alpern, Amanda Montalbano

**Affiliations:** 1 Department of Pediatrics, Johns Hopkins University School of Medicine, 1800 Orleans St, Suite G-1509, Baltimore, MD 21287 USA; 2 Department of Emergency Medicine, Beth Israel Deaconess Medical Center, 330 Brookline Avenue, Boston, MA 02215 USA; 3 Department of Analytics, Children’s Hospital Association, 16011 College Blvd, Lenexa, Kansas, 66219 USA; 4 Division of Emergency Medicine, Department of Pediatrics, Ann and Robert H. Lurie Children’s Hospital of Chicago, 225 East Chicago Ave, Box 62, Chicago, IL 60611 USA; 5 Department of Pediatrics, George Washington University School of Medicine and Health Sciences, 111 Michigan Ave NW, Washington, DC, 20010 USA; 6 Department of Pediatrics, Children’s Mercy Hospitals and Clinics, 20300 East Valley View Pkwy, Independence, MO 64057 USA

**Keywords:** Ambulatory care, Emergency care, Managed care administration, Capitation/payment methods, Urgent care, Acute/subacute care, Medicare/Medicaid, Accountable care organizations

## Abstract

**Background:**

In the United States (US), Medicaid capitated managed care costs are controlled by optimizing patients’ healthcare utilization. Adults in capitated plans utilize primary care providers (PCP) more than emergency departments (ED), compared to fee-for-service (FFS). Pediatric data are lacking. We aim to determine the association between US capitated and FFS Medicaid payment models and children’s outpatient utilization.

**Methods:**

This retrospective cohort compared outpatient utilization between two payment models of US Medicaid enrollees aged 1–18 years using Truven’s 2014 Marketscan Medicaid database. Children enrolled > 11 months were included, and were excluded for eligibility due to disability/complex chronic condition, lack of outpatient utilization, or provider capitation penetration rate < 5% or > 95%. Negative binomial and logistic regression assessed relationships between payment model and number of visits or odds of utilization, respectively.

**Results:**

Of 711,008 children, 66,980(9.4%) had FFS and 644,028(90.6%) had capitated plans. Children in capitated plans had greater odds of visits to urgent care, PCP-acute, and PCP-well-child care (aOR 1.21[95%CI 1.15–1.26]; aOR 2.07[95%CI 2.03–2.13]; aOR 1.86 [95%CI 1.82–1.91], respectively), and had lower odds of visits to EDs and specialty care (aOR 0.82 [95%CI 0.8–0.83]; aOR 0.61 [95%CI 0.59–0.62], respectively), compared to FFS.

**Conclusions:**

The majority of children in this US Medicaid population had capitated plans associated with higher utilization of acute care, but increased proportion of lower-cost sites, such as PCP-acute visits and UC. Health insurance programs that encourage capitated payment models and care through the PCP may improve access to timely acute care in lower-cost settings for children with non-complex chronic conditions.

## Background

Since the United States’ (US) Affordable Care Act (ACA) of 2010 and Medicare Access and Children’s Health Insurance Program (CHIP) Reauthorization Act (MACRA) of 2015, enrollment of children in Medicaid or CHIP has grown to historically high rates [1–3]. Most newly insured children are covered under comprehensive managed care organizations (MCO), which utilize capitated payment models [4]. Under capitated payment models, state Medicaid agencies and MCOs agree to a fixed payment, per member, per month, to control health costs and coordinate care [5]. The MCO then pays providers on either a capitated or FFS basis [6, 7]. Providers are encouraged to control costs by limiting low-value tests and treatments, advocating for prevention, and promoting children to seek care at cost-effective venues, preferably coordinated through the primary care provider (PCP) [8]. The enrollment of children into a MCO or fee-for-service (FFS) plan is varied by state policies, and includes factors such as plan availability, geography, disability or complex care needs, or enrollee choice. Children in FFS Medicaid plans are associated with greater expenditures [9] and in concordance with this, the portion of children enrolled in capitated MCOs are rising and FFS plans declining [4, 10, 11]. As public policy advocates for children, pediatric providers should have awareness of whether capitated payment models are associated with children’s use of cost-effective locations, such as the PCP.

The effect of Medicaid payment models on children’s healthcare utilization was studied 2–3 decades ago, when the enrollment and management of Medicaid managed care differed greatly from today’s landscape. These studies found Medicaid managed care prepaid plans (akin to today’s capitated plans) were associated with no change in well child visits, and no change or decreased ED visits [12–14]. Since then, studies of Medicaid payment models in children have been limited to primary or preventative care, or limited to children with special healthcare needs or based on self-report [15–17]. Recent studies on outpatient utilization in the commercially insured population include urgent care (UC), however data on UC utilization in the Medicaid population are lacking [18, 19]. UC visits are one example of possible healthcare cost savings, as they are significantly less expensive than ED visits for comparable illnesses, and are an increasingly used venue for low acuity conditions [18, 20]. What remains unknown is the association of current payment models on outpatient care-seeking behavior in children, after the major healthcare policy changes associated with the ACA in 2010 and the 2014 Medicaid expansion. This study aims to describe the association of current Medicaid payment models with children’s utilization of outpatient care.

## Methods

### Study design and setting

We conducted a retrospective cohort study comparing outpatient utilization between two payment models (capitated versus FFS) of US Medicaid enrollees aged 1 to 18 years using Truven - IBM Watson Health’s (New York, NY) Marketscan Medicaid claims database for the year 2014. The database contains inpatient, outpatient, long-term care, and retail pharmacy claims from eleven de-identified, geographically-varied states. The states in the 2014 Marketscan database are representative of the overall Medicaid population and ranged from having 7 to 100% capitated enrollees (correspondence with Truven – Watson IBM Health), but no state identifiers are present in the database.

We included non-chronically ill children that were enrolled in Medicaid for at least 11 months of the study year. We defined non-chronically ill children as those without medical complexity or disability, though some patients may have multiple non-complex medical conditions (e.g. allergic rhinitis). We excluded enrollees with a complex chronic condition (CCC), as defined by Feudtner et al. [21], due to the disproportionate use of healthcare services by chronically-ill children. We excluded enrollees eligible for Medicaid due to a disability because of state-to-state variability in preferentially assigning these children to a fee-for-service payment model [8, 22]. Children aged less than 12 months were excluded due to inability to determine sustained enrollment for 11 of 12 study months and therefore inability to track utilization during their life.

We accounted for provider level practice influences by calculating the capitation penetrance rate for each provider. In this database, the managed care status for each of the enrollees that is seen by (i.e. has a claim by) a provider is aggregated to produce a value (i.e. 0–100% capitation penetration rate) for that provider. With this data, we excluded enrollees who saw any provider with a capitation penetration rate of less than 5% or more than 95%, or who did not see at least 45 Medicaid enrollees in the study year. Extremes in capitation penetrance rate were excluded to reduce bias arising from providers who may be influenced in their referral patterns due to penetrance of their payer type, and to reduce bias from states that have exclusively one type of payment model.

### Ethics

This study was reviewed and approved as non- human subjects research by the Institutional Review Board at Children’s Mercy Kansas City.

### Population and visit characteristics

Patient age was calculated as of December 31, 2014. We also report sex and race/ethnicity (White, Black, Hispanic, Other). To compare disease burden between the two payment groups in our cohort, we identified and characterized non-complex chronic medical conditions. The presence and number of non-complex chronic medical conditions (e.g. allergic rhinitis) and their organ systems were identified using the Agency for Healthcare Research and Quality’s (AHRQ) Chronic Condition Indicator. AHRQ’s Chronic Condition Indicator is a publicly available diagnosis-based classification system that identifies International Classification of Diseases, Ninth Revision, Clinical Modification (ICD-9-CM) codes as chronic or not chronic, as well as the affected organ system (e.g. cardiac, endocrine, hematology, etc.) [23].

The outpatient visit sites of ED, UC, PCP acute visit, PCP well-child visit, specialty care were classified based on the coded location of services [24] and Truven’s proprietary service subcategory provided in the claims data. Truven’s database classifies specialty care to ancillary or specialty health services that are outside of the scope of a PCP visit (e.g. medical or surgical sub-specialists, optometry, mental health, etc.). Retail based clinics were not included, due to an exceedingly small sample in this category (less than 100 retail clinic visits in the entire database). PCP well-child visits were differentiated from PCP acute visits using ICD-9 or Current Procedural Terminology codes, based on a previously developed algorithm [25].

### Statistical analysis

Differences in demographics, clinical characteristics, and utilization between capitated and FFS enrollees were determined by chi-squared tests of association. The relationship between payment model and number of visits in each of the care settings was assessed with negative binomial regression. Logistic regression assessed the adjusted relationship between payment model and the odds of having any utilization or rate ratio of visits in the different care settings. Models were adjusted for age group (1–2, 3–5, 6–12, 13–18 years), sex, race/ethnicity, number of non-complex medical conditions, and non-complex medical condition organ systems. All analyses were performed with SAS 9.4 (SAS Institute, Cary, NC). *P*-values less than 0.05 were considered statistically significant.

## Results

**Fig. 1:**
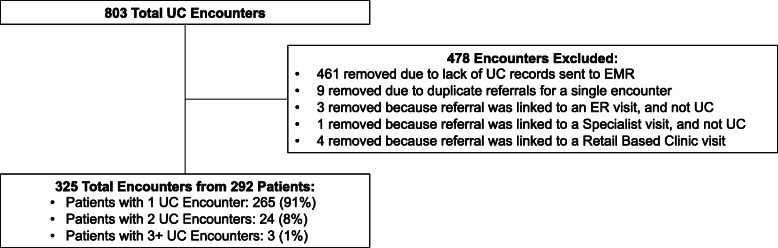
Flow chart of Medicaid enrollees included for analysis

Our study population included 711,008 total children, of which 66,980 (9.4%) had FFS plans and 644,028 (90.6%) were in capitated plans. A diagram of enrollees included for analysis is displayed in Fig. 1. Most children in both groups were in the 6- to 12-year-old age range, though the capitated group had more toddler and pre-school aged children while the FFS group had more adolescents, as shown in Table 1. The capitated group had a significantly higher proportion of minority children (46.6% of children in the capitated group were black vs. 24.5% of the FFS group; *p* < 0.001). The FFS group contained more children with three or more non-complex medical conditions compared those with capitated care (11.6% vs 9.0%; *p* < 0.001). The prevalence of non-complex medical conditions categorized by organ systems was not significantly different in the two populations for 10 of 22 organ system categories, shown in Additional file 1. The four most common medical conditions in both groups were allergic rhinitis, vision defects, ADD & ADHD, and asthma. Supplemental data on the association of outpatient care setting with age, sex, race/ethnicity, and number of non-complex medical conditions is shown in Additional file 2.

**Table 1:** Demographics of study population stratified by payment model. ^**a**^ The presence and number of non-complex medical conditions were identified using the Agency for Healthcare Research and Quality’s (AHRQ) Chronic Condition Indicator, a publicly available diagnosis-based classification system that identifies International Classification of Diseases, Ninth Revision, Clinical Modification (ICD-9-CM) codes as chronic or not chronic [23]

Characteristic	Total	Fee for Service	Capitated	***P***-value
	***N*** = 711,008 (100%)	***N*** = 66,980 (9.4%)	***N*** = 644,028 (90.6%)	
**Age (years)**				< 0.001
1–2	87,730 (12.3)	7630 (11.4)	80,100 (12.4)	
3–5	136,396 (19.2)	9565 (14.3)	126,831 (19.7)	
6–12	304,444 (42.8)	29,284 (43.7)	275,160 (42.7)	
13–18	182,438 (25.7)	20,501 (30.6)	161,937 (25.1)	
**Sex**				0.57
Male	362,424 (51.0)	34,212 (51.1)	328,212 (51.0)	
Female	348,584 (49.0)	32,768 (48.9)	315,816 (49.0)	
**Race/Ethnicity**				< 0.001
White	312,358 (43.9)	45,643 (68.1)	266,715 (41.4)	
Black	316,814 (44.6)	16,392 (24.5)	300,422 (46.6)	
Hispanic	44,335 (6.2)	1880 (2.8)	42,455 (6.6)	
Other	37,501 (5.3)	3065 (4.6)	34,436 (5.3)	
**Number of Non-Complex Medical Conditions**				< 0.001
0	368,071 (51.8)	34,576 (51.6)	333,495 (51.8)	
1	191,631 (27.0)	17,286 (25.8)	174,345 (27.1)	
2	85,889 (12.1)	7381 (11.0)	78,508 (12.2)	
3+	65,417 (9.2)	7737 (11.6)	57,680 (9.0)	

Enrollees in capitated plans had a significantly greater proportion of > 1 visits per year to EDs, UCs, and PCP for acute care, and significantly smaller proportion of > 1 specialty care visits, compared to children in FFS plans (all *p* < 0.001). (Table 2) The highest rate of visits per 100 enrollees was for children in capitated plans to the PCP for acute care (155 visits per 100 enrollees), which was nearly 4-fold greater than their rate of ED visits (39 visits per 100 enrollees), and nearly 3-fold greater than the rate of PCP-acute care in FFS plans (55 visits per 100 enrollees). The highest rate of visits for children in FFS plans were to specialty care (177 visits per 100 enrollees). (Table 2).

**Table 2:** Rate and frequency of site utilization listed by insurance type. PCP-Well Child Visits are excluded from this table because recommended visits per year vary by age

Care Setting	Total	Fee for Service	Capitated	***P***-value
**Emergency Department (ED)**				
ED Visits per 100 Enrollees	39	34	39	< 0.001
Number of ED Visits, N (%)				< 0.001
0	532,109 (74.8)	52,562 (78.5)	479,547 (74.5)	
1	120,067 (16.9)	9550 (14.3)	110,517 (17.2)	
2	37,411 (5.3)	3021 (4.5)	34,390 (5.3)	
3	12,750 (1.8)	1047 (1.6)	11,703 (1.8)	
4+	8671 (1.2)	800 (1.2)	7871 (1.2)	
**Urgent Care (UC)**				
UC Visits per 100 Enrollees	9	8	9	< 0.001
Number of UC Visits, N (%)				< 0.001
0	671,694 (94.5)	63,499 (94.8)	608,195 (94.4)	
1	26,694 (3.8)	2396 (3.6)	24,298 (3.8)	
2	7664 (1.1)	700 (1.0)	6964 (1.1)	
3+	4956 (0.7)	385 (0.6)	4571 (0.7)	
**Primary Care (PCP)- Acute Visits**				
PCP-Acute Visits per 100 Enrollees	145	55	155	< 0.001
Number of PCP-Acute Visits, N (%)				< 0.001
0	330,115 (46.4)	51,561 (77.0)	278,554 (43.3)	
1	150,200 (21.1)	7213 (10.8)	142,987 (22.2)	
2	86,952 (12.2)	3502 (5.2)	83,450 (13.0)	
3	52,002 (7.3)	1838 (2.7)	50,164 (7.8)	
4	32,308 (4.5)	1085 (1.6)	31,223 (4.8)	
5+	59,431 (8.4)	1781 (2.7)	57,650 (9.0)	
**Specialty Care Visits**				
Specialty Care Visits per 100 Enrollees	142	170	139	< 0.001
Number of Specialty Care Visits, N (%)				< 0.001
0	396,711 (55.8)	30,911 (46.1)	365,800 (56.8)	
1	149,751 (21.1)	16,643 (24.8)	133,108 (20.7)	
2	64,643 (9.1)	7458 (11.1)	57,185 (8.9)	
3	33,366 (4.7)	4147 (6.2)	29,219 (4.5)	
4	19,546 (2.7)	2497 (3.7)	17,049 (2.6)	
5+	46,991 (6.6)	5324 (7.9)	41,667 (6.5)	
**Total Visits per 100 Enrollees**	335	266	342	

**Fig. 2:**
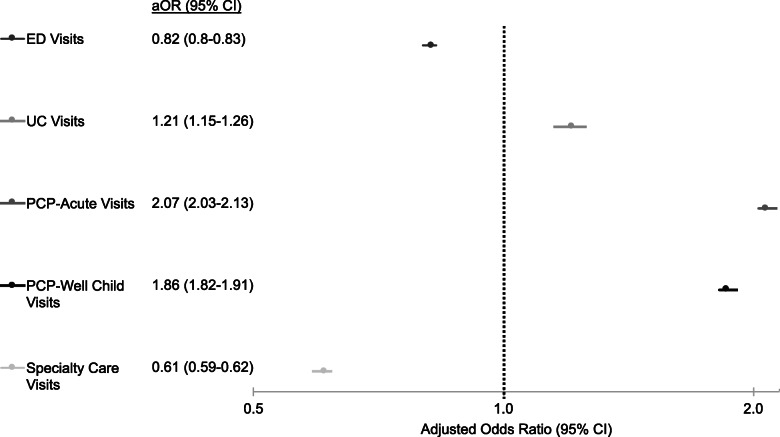
Adjusted odds ratio (aOR) of utilization of outpatient care settings by children in capitated plans. Children in FFS plans were the reference group: FFS aOR (95% CI): 1 (0,0)

When adjusting for demographics and non-complex medical conditions, the odds were 21, 107, and 86% higher for children in capitated plans to visit UC, PCP-acute care, and PCP-well-child care (aOR 1.21, 95%CI 1.15–1.26; aOR 2.07, 95%CI 2.03–2.13; aOR 1.86, 95%CI 1.82–1.91, respectively), noted in Fig. 2. In contrast, among children in capitated plans, the odds were 18 and 39% lower for ED or specialty care visits (aOR 0.82, 95%CI 0.8–0.83; aOR 0.61, 95%CI 0.59–0.62, respectively). The model adjusting for number of visits to each care setting showed similar results.

## Discussion

Our results suggest children in capitated plans have patterns of outpatient utilization notable for greater use of urgent care and PCP-acute and well-child care, with lower odds of visits to EDs and specialty care. Although capitated enrollees had higher rates of overall outpatient utilization, regression analysis showed they were more likely to seek care in less costly locations (UC and PCP). In contrast, children in FFS plans were more likely to seek care at costlier sites (ED and specialty care).

Studies of disabled children and of adults found similar trends. Previous literature commented that disabled children in capitated plans had easier access to primary care and better coordination of emergency care, compared to those in FFS plans [15, 16]. Studies of adults in capitated plans, performed after the ACA in 2010 and the 2014 Medicaid expansion, reported lower rates of ED utilization and higher rates of PCP visits [4, 26, 27]. However, other reports noted a marginal increase [28, 29], or no change in rate of ED visits [30]. Recent years have seen increased enrollment in capitated MCOs, declining enrollment in FFS plans [31], but also declining enrollment of children overall [32]. While longitudinal, nationwide studies of children are still warranted to ensure MCO payment models are indeed leading to cost-effective healthcare utilization, our data supports a trend of lower-cost sites of utilization by children with capitated MCOs. Policy makers and health systems should continue to support healthcare coverage of children and support programs that use capitated payment models with care through the PCP to improve timely access to lower-cost acute care settings for children with non-complex chronic conditions.

Our analysis also found that children in capitated plans had increased frequency of utilization (3, 4, or 5+ visits per year) of all acute care sites (ED, UC, PCP), compared to those in FFS plans. If children in capitated plans are high-frequency utilizers of healthcare, despite visiting lower-cost care settings (e.g. UC, PCP), the high volume of acute care visits may negate the financial benefit of capitated payment models. However, this trend of high acute care utilization by capitated enrollees may be a transient finding during a time of health insurance change. The phenomenon that pent-up demand plus increased access to healthcare leads to increased utilization was described among newly insured children after Oregon’s 2009–2010 CHIP expansion, and in adults after the 2014 Medicaid expansion under the ACA [17, 33]. Expansion programs that increase parents’ Medicaid eligibility (such as the 2014 Medicaid expansion and Oregon’s 2008 Medicaid expansion) have been associated with a ‘welcome mat’ effect on families, leading to increased enrollment in children [34–36]. Since these landmark changes in health insurance, children have had increased enrollment rates in Medicaid or CHIP, and likewise increased access to healthcare [1]. It’s unclear whether our finding of high acute care utilization by capitated enrollees reflects pent-up demand of newly insured children or a well-established medical home model in this cohort. This theory merits validation with longitudinal data.

Strategies on cost-containment based on site of healthcare delivery should focus on supporting the PCP even for FFS plans [37]. Past work supports the notion that PCPs and patients in a capitated system experience improved first-contact accessibility and an improved availability of services [38, 39]. However, when the PCP is not available, strategies to provide access to care at the most cost-effective site should be promoted. UC comprised a small portion of acute visits in both payment models, whereas ED visits were more common. Since UC offers cost-savings compared to similar ED visits, secondary cost-containment strategies may focus on shifting low acuity ED visits to UC [20]. The largest portion of acute care visits by children in capitated plans occurred at the PCP, and the smallest portion of acute care visits were to UC. This underscores the important role of PCPs in serving as the hub of the medical neighborhood, for children with acute or chronic illnesses.

Other explanations for high utilization for acute care visits in capitated plans, compared to FFS, may arise from influences other than that of the primary care provider. First, previous studies have shown that low-income families enrolled in health insurance plans with cost-sharing, compared to plans without cost-sharing, had decreased episodes of primary and emergency care, and reduced ambulatory expenses by up to one-third [40, 41]. The health implications of this reduction in acute care are not known currently. Cost-sharing still exists in Medicaid and CHIP but there is wide state-to-state variability in the structure of cost-sharing [2]. Second, minority races have been associated with some disparities in pediatric healthcare utilization, such as lack of a usual source of care prior to CHIP enrollment and receipt of fewer primary care services, while other studies show no healthcare disparities amongst race [42, 43]. Our study found a higher rate of minorities (Black, Hispanic, or Other) enrolled in capitated plans, compared to FFS, and greater odds of ED visits in black enrollees (Additional file 2). Unadjusted rates of capitated plans showed higher numbers of ED visits, but when controlling for demographics and non-complex medical conditions adjusted odds of ED visits were lower in the capitated group. It’s unclear whether race influenced utilization, or these findings are an effect of baseline race/ethnicity disparities [42]. Alternatively, the differences in races enrolled in the two payment plans may reflect state-level population characteristics and local availability of Medicaid payment models. The influences on patient behavior and healthcare utilization are complex and multi-factorial, and remain an ongoing area of research. Furthermore, utilization is merely one facet that contributes to the success of a payment model. Evaluation of current payment models must incorporate a multitude of factors beyond utilization, such as appropriateness of use, race/ethnicity influences, equitable access to health care, and health outcomes. Capitated plans can also influence healthcare choices by changing how providers deliver care (as when primary care providers are themselves capitated), through network size (to higher or lower provider payment rates than FFS Medicaid), or through direct effects on enrollees (e.g. through prevention, information provision, or ED copays where permitted). Lastly, this cross-sectional, observational study does not determine causality related to the utilization patterns of children between the FFS and capitated groups. However, observable characteristics of the FFS group included more non-complex medical conditions. Therefore, if these characteristics would require increased medical attention, we would expect more acute care visits among FFS, rather than fewer. Nevertheless, this comprehensive, multi-state database offered rich information to characterize outpatient visits in the pediatric Medicaid population.

### Limitations

Limitations of this study include generalizability arising from our choice to exclude children whose providers see exclusively one type of payment model (i.e. < 5 or > 95% capitation penetration rate), excluding > 2 million enrollees; however, this was necessary to alleviate the predominant practice patterns within regions with a single payment model and made the sample population as comparable as possible. This exclusion reduces the generalizability of the results, as children in the final sample were likely less geographically diverse, were a higher percentage Black, had more chronic conditions, and had more utilization. A known limitation of Medicaid databases is the high turnover of enrollment. We included enrollees with > 11 months of continuous enrollment in order to capture a comprehensive view of outpatient visits in this cohort, consequently excluding another 1.2 million enrollees. The implications of a large number of enrollee exclusions is a potential bias of the outpatient utilization patterns noted in this study. Another limitation of de-identified US Medicaid databases is lack of state-specific Medicaid policies and lack of provider-level utilization (since claims are listed at the enrollee level), which limits our ability to draw conclusions or account for trends in utilization at the state or provider/practice level. Other facets unable to be determined from this database is information on specific plan types (e.g. primary care case management (PCCM)), or modes of provider reimbursement. Therefore our estimate is an average effect of patients in capitated plans whose providers are paid on a capitated basis and a FFS basis. A potential confounder that is not accounted for in this database includes urban versus rural geography of enrollees, or proximity of available in-network providers. If Medicaid managed care is more common in urban areas or has more available in-network providers, and urban dwellers have greater healthcare use and access [44], then geography differences may contribute to utilization disparities in this study. We controlled for high illness-severity by excluding disabled children or with complex chronic conditions, but given the constraints of this database we do not have a reliable means to quantify severity of disease in the remaining study population. Despite applying a comprehensive classification system to exclude complex chronic conditions, some chronic conditions including mental health illnesses may not have been identified in the study population. As such it is possible that some of the differences in utilization we noted in this study are, in part, attributed to differences in severity of non-complex chronic conditions or differences in complex chronic or mental health conditions associated with increased morbidity. While this study evaluates pediatric outpatient utilization, we are unable to draw conclusions on appropriateness of use, unmet needs, or health outcomes of these children. Finally, the rapidly changing climate of healthcare in the United States makes it difficult to predict whether the patterns from this single-year study will remain in the future.

## Conclusion

This study reports the outpatient utilization patterns of children with different Medicaid insurance payment models in the US. Children in capitated plans were more likely to visit the medical home and less costly locations (UC, PCP) and were less likely to visit costlier sites (ED, specialty care). This study included data from 2014. Since 2014, Medicaid continues to experience shifts in payment models, shifts in numbers of insured children, and potentially changes in access to care [4, 8, 10, 32]. Longitudinal, nationwide studies of children are warranted to ensure MCO payment models are indeed leading to cost-effective healthcare utilization in the pediatric population. Given the trend of lower-cost sites of utilization by children with capitated MCOs, policy makers and health systems should continue to support health insurance programs for children that use capitated payment models and care through the PCP to improve timely access to lower-cost acute care settings for children with non-complex chronic conditions.

## Supplementary information

**Additional file 1 **Prevalence of non-complex medical condition organ systems and 4 most common non-complex medical conditions, by insurance type. ^**a**^ The presence and number of non-complex medical conditions and their organ systems were identified using the Agency for Healthcare Research and Quality’s (AHRQ) Chronic Condition Indicator, a publicly available diagnosis-based classification system that identifies International Classification of Diseases, Ninth Revision, Clinical Modification (ICD-9-CM) codes as chronic or not chronic, as well as the affected organ system [23].

**Additional file 2.** Adjusted Odds Ratio of demographics and non-complex medical conditions by outpatient care setting. Odds Ratios are adjusted for age, sex, race/ethnicity, and number of non-complex medical conditions (excluding the co-variate of interest). ^a^ The presence and number of non-complex medical conditions were identified using the Agency for Healthcare Research and Quality’s (AHRQ) Chronic Condition Indicator, a publicly available diagnosis-based classification system that identifies International Classification of Diseases, Ninth Revision, Clinical Modification (ICD-9-CM) codes as chronic or not chronic [23].

## Data Availability

The datasets used and/or analyzed during the current study are available from the corresponding author on reasonable request.

## Abbreviations

CHIP: Children’s Health Insurance Program
PCP: Primary care provider
FFS: Fee-for-service
MCO: Managed care organization
ED: Emergency Department
UC: Urgent Care
aOR: Adjusted odds ratio
RR: Rate ratio
US: United States
