# Supplementary material for: Pediatric outpatient utilization by differing Medicaid payment models in the United States
Source: BMC Health Serv Res. 2020 Jun 12;20:532. doi: 10.1186/s12913-020-05409-w (PMC7291721; doi:10.1186/s12913-020-05409-w)
Supplement: Supplementary file 1 — Additional file 1 Prevalence of non-complex medical condition organ systems and 4 most common non-complex medical conditions, by insurance type. a The presence and number of non-complex medical conditions and their organ systems were identified using the Agency for Healthcare Research and Quality’s (AHRQ) Chronic Condition Indicator, a publicly available diagnosis-based classification system that identifies International Classification of Diseases, Ninth Revision, Clinical Modification (ICD-9-CM) codes as chronic or not chronic, as well as the affected organ system [23]. [file 12913_2020_5409_MOESM1_ESM.docx]

| **Characteristic** | | **Total** | **Fee for Service** | **Capitated** | **p-value** |
| --- | --- | --- | --- | --- | --- |
|  |  | **N = 711,008 (100%)** | **N = 66,980**  **(9.4%)** | **N = 644,028 (90.6%)** |  |
| **Non-Complex Medical Condition Organ System** | | | |  |  |
|  | Infection | 550 (0.1) | 44 (0.1) | 506 (0.1) | 0.254 |
|  | Cancer | 22 (0.0) | 1 (0.0) | 21 (0.0) | 0.717 |
|  | Endocrine | 5,895 (0.8) | 652 (1.0) | 5,243 (0.8) | <0.001 |
|  | Hematology | 1,021 (0.1) | 94 (0.1) | 927 (0.1) | 0.815 |
|  | Mental | 127,499 (17.9) | 14,387 (21.5) | 113,112 (17.6) | <0.001 |
|  | Neuro | 11,468 (1.6) | 1,267 (1.9) | 10,201 (1.6) | <0.001 |
|  | Cardiac | 3,767 (0.5) | 433 (0.6) | 3,334 (0.5) | <0.001 |
|  | Respiratory | 153,832 (21.6) | 115,58 (17.3) | 142,274 (22.1) | <0.001 |
|  | Digestive | 14,422 (2.0) | 1,298 (1.9) | 13,124 (2.0) | 0.081 |
|  | Urogenital | 19,270 (2.7) | 1,961 (2.9) | 17,309 (2.7) | <0.001 |
|  | Pregnancy | 404 (0.1) | 48 (0.1) | 356 (0.1) | 0.090 |
|  | Skin | 30,407 (4.3) | 1,560 (2.3) | 28,847 (4.5) | <0.001 |
|  | Musculoskeletal | 7,023 (1.0) | 715 (1.1) | 6,308 (1.0) | 0.028 |
|  | Trauma/complications | 6 (0.0) | 1 (0.0) | 5 (0.0) | 0.448 |
|  | Aftercare/screening | 2 (0.0) | 1 (0.0) | 1 (0.0) | 0.180 |
|  | Nutrition | 1,817 (0.3) | 204 (0.3) | 1,613 (0.3) | 0.008 |
|  | Immune | 52 (0.0) | 4 (0.0) | 48 (0.0) | 1.000 |
|  | Renal | 183 (0.0) | 19 (0.0) | 164 (0.0) | 0.656 |
|  | Oral/dental | 943 (0.1) | 141 (0.2) | 802 (0.1) | <0.001 |
|  | Hearing/ear | 12,367 (1.7) | 1,186 (1.8) | 11,181 (1.7) | 0.515 |
|  | Vision/eye | 71,894 (10.1) | 10,864 (16.2) | 61,030 (9.5) | <0.001 |
|  | Metabolic | 28,698 (4.0) | 1,323 (2.0) | 27,375 (4.3) | <0.001 |
| **Common Non-Complex Medical Conditions** | | |  |  |  |
|  | Allergic Rhinitis | 103,214 (14.5) | 7,531 (11.2) | 95,683 (14.9) | <0.001 |
|  | Vision defects | 69,943 (9.8) | 10,715 (16.0) | 59,228 (9.2) | <0.001 |
|  | ADD & ADHD | 60,029 (8.4) | 6,871 (10.3) | 53,158 (8.3) | <0.001 |
|  | Asthma | 65,056 (9.1) | 4,675 (7.0) | 60,381 (9.4) | <0.001 |
