# Supplementary material for: Pediatric outpatient utilization by differing Medicaid payment models in the United States
Source: BMC Health Serv Res. 2020 Jun 12;20:532. doi: 10.1186/s12913-020-05409-w (PMC7291721; doi:10.1186/s12913-020-05409-w)
Supplement: Supplementary file 2 — Additional file 2. Adjusted Odds Ratio of demographics and non-complex medical conditions by outpatient care setting. Odds Ratios are adjusted for age, sex, race/ethnicity, and number of non-complex medical conditions (excluding the co-variate of interest). a The presence and number of non-complex medical conditions were identified using the Agency for Healthcare Research and Quality’s (AHRQ) Chronic Condition Indicator, a publicly available diagnosis-based classification system that identifies International Classification of Diseases, Ninth Revision, Clinical Modification (ICD-9-CM) codes as chronic or not chronic [23]. [file 12913_2020_5409_MOESM2_ESM.docx]

|  |  | **ED Visits** | | **UC Visits** | | **PCP- Acute Visits** | | **PCP- Well Child Visits** | | **Specialty Care Visits** | |
| --- | --- | --- | --- | --- | --- | --- | --- | --- | --- | --- | --- |
|  |  | **aOR** | **95% CI** | **aOR** | **95% CI** | **aOR** | **95% CI** | **aOR** | **95% CI** | **aOR** | **95% CI** |
| **Age (years)** | |  |  |  |  |  |  |  |  |  |  |
|  | 1-2 | Ref |  | Ref |  | Ref |  | Ref |  | Ref |  |
|  | 3-5 | 0.60 | (0.59, 0.61) | 0.92 | (0.887, 0.95) | 0.61 | (0.60, 0.63) | 0.27 | (0.27, 0.28) | 0.91 | (0.89, 0.92) |
|  | 6-12 | 0.37 | (0.37, 0.38) | 0.75 | (0.72, 0.77) | 0.35 | (0.34, 0.36) | 0.10 | (0.09, 0.1) | 1.19 | (1.17, 1.21) |
|  | 13-18 | 0.42 | (0.41, 0.43) | 0.70 | (0.68, 0.73) | 0.29 | (0.29, 0.3) | 0.08 | (0.08, 0.08) | 1.21 | (1.18, 1.23) |
| **Sex** | |  |  |  |  |  |  |  |  |  |  |
|  | Male | 1.04 | (1.03, 1.06) | 0.97 | (0.95, 0.99) | 0.91 | (0.90, 0.92) | 1.03 | (1.02, 1.04) | 0.94 | (0.93, 0.95) |
|  | Female | Ref |  | Ref |  | Ref |  | Ref |  | Ref |  |
| **Race/Ethnicity** | |  |  |  |  |  |  |  |  |  |  |
|  | White | Ref |  | Ref |  | Ref |  | Ref |  | Ref |  |
|  | Black | 1.30 | (1.23, 1.31) | 0.61 | (0.6, 0.63) | 0.52 | (0.51, 0.52) | 1.08 | (1.07, 1.09) | 0.84 | (0.83, 0.85) |
|  | Hispanic | 0.90 | (0.88, 0.92) | 0.69 | (0.66, 0.72) | 1.08 | (1.05, 1.10) | 1.28 | (1.25, 1.31) | 1.06 | (1.04, 1.08) |
|  | Other | 0.69 | (0.67, 0.70) | 0.75 | (0.72, 0.79) | 1.00 | (0.98, 1.03) | 1.32 | (1.29, 1.35) | 0.65 | (0.64, 0.67) |
| **Number of Non-Complex Medical Conditions** | | | | |  |  |  |  |  |  |  |
|  | 0 | Ref |  | Ref |  | Ref |  | Ref |  | Ref |  |
|  | 1 | 1.12 | (1.09, 1.14) | 1.05 | (1.00, 1.09) | 1.45 | (1.42, 1.5) | 1.23 | (1.2, 1.25) | 1.46 | (1.42, 1.49) |
|  | 2 | 1.29 | (1.25, 1.33) | 1.10 | (1.03, 1.17) | 1.94 | (1.86, 2.01) | 1.39 | (1.35, 1.44) | 1.96 | (1.89, 2.03) |
|  | 3+ | 1.65 | (1.58, 1.73) | 1.23 | (1.13, 1.34) | 2.42 | (2.3, 2.55) | 1.39 | (1.33, 1.45) | 2.64 | (2.52, 2.77) |
